# Bioactive Compounds and Antioxidant Activity of *Boletus edulis*, *Imleria badia*, *Leccinum scabrum* in the Context of Environmental Conditions and Heavy Metals Bioaccumulation

**DOI:** 10.3390/molecules30153277

**Published:** 2025-08-05

**Authors:** Zofia Sotek, Katarzyna Malinowska, Małgorzata Stasińska, Ireneusz Ochmian

**Affiliations:** 1 Institute of Marine and Environmental Sciences, University of Szczecin, Adama Mickiewicza 16 Street, 70-383 Szczecin, Poland; 2 Department of Bioengineering, West Pomeranian University of Technology in Szczecin, Słowackiego 17 Street, 71-434 Szczecin, Poland; katarzyna.malinowska@zut.edu.pl; 3 Department of Horticulture, West Pomeranian University of Technology in Szczecin, Słowackiego 17 Street, 71-434 Szczecin, Poland; ireneusz.ochmian@zut.edu.pl

**Keywords:** phenolic compounds, glucans, organic acid, antioxidant properties, edible mushrooms, microclimate and soil impact

## Abstract

Wild edible mushrooms are increasingly recognised for their nutritional and therapeutic potential, owing to their richness in bioactive compounds and antioxidant properties. This study assessed the chemical composition, antioxidant capacity, and bioaccumulation of heavy metals (Cd, Pb, Ni) in *Boletus edulis*, *Imleria badia*, and *Leccinum scabrum* collected from two forested regions of north-western Poland differing in anthropogenic influence and soil characteristics. The analysis encompassed structural polysaccharides (β- and α-glucans, chitin), carotenoids, L-ascorbic acid, phenolic and organic acids. *B. edulis* exhibited the highest β-glucan and lycopene contents, but also the greatest cadmium accumulation. *I. badia* was distinguished by elevated ascorbic and citric acid levels and the strongest DPPH radical scavenging activity, while *L. scabrum* showed the highest ABTS and FRAP antioxidant capacities and accumulated quinic acid and catechin. Principal component analysis indicated strong correlations between antioxidant activity and phenolic acids, while cadmium levels were inversely associated with antioxidant potential and positively correlated with chitin. Although all metal concentrations remained within EU food safety limits, *B. edulis* showed consistent cadmium bioaccumulation. From a practical perspective, the results highlight the importance of species selection and sourcing location when considering wild mushrooms for consumption or processing, particularly in the context of nutritional value and contaminant load. Importantly, regular or excessive consumption of *B. edulis* may result in exceeding the tolerable weekly intake (TWI) levels for cadmium and nickel, which warrants particular attention from a food safety perspective. These findings underscore the influence of species-specific traits and environmental conditions on mushroom biochemical profiles and support their potential as functional foods, provided that metal contents are adequately monitored.

## 1. Introduction

Wild-growing mushrooms have long constituted an integral part of culinary heritage and socio-cultural traditions across many regions of the world [1,2,3]. In Poland, approximately 100,000 tonnes of wild forest mushrooms are harvested annually, underscoring their culinary, cultural, and economic importance [4]. Among the most highly valued species in Poland and throughout Europe is *Boletus edulis*, which features prominently in traditional dishes prepared from foraged mushrooms [5]. By contrast, in North America, species such as *Cantharellus* spp. and *Morchella* spp. are more commonly collected [6], although mushroom consumption there largely relies on commercially cultivated species, especially *Agaricus bisporus* [7]. Globally, mushroom production has grown substantially, reaching approximately 44 million tonnes in 2021 [8].

Beyond their gastronomic significance, mushrooms are increasingly recognised for their favourable nutritional profiles and potential health-promoting effects. These benefits are attributed to a wide array of bioactive compounds—such as polysaccharides, phenolics, vitamins, and minerals—which possess anticancer, anti-inflammatory, and antioxidant properties [9]. Among these, β-glucans are particularly notable for their immunomodulatory capacity, as they stimulate macrophages, natural killer (NK) cells, and T lymphocytes. They have also been associated with reductions in blood cholesterol levels and the regulation of glycaemia [10,11]. Phenolic compounds, including flavonoids and phenolic acids, play a crucial role in mitigating oxidative stress by scavenging reactive oxygen species (ROS), thereby potentially lowering the risk of chronic diseases such as cardiovascular and neurodegenerative disorders [12,13,14].

The antioxidant capacity of mushrooms has been confirmed in extracts from species such as *Boletus edulis* and *Cantharellus cibarius*, which contain gallic acid, p-coumaric acid, and quercetin—key contributors to radical scavenging activity [15]. Antioxidant properties are also attributed to carotenoids (e.g., β-carotene) and to L-ascorbic acid [16,17]. Organic acids such as malic, citric, and fumaric acid not only enhance flavour and product stability but also exhibit antimicrobial activity and may support metabolic and digestive functions [18]. Their health-promoting potential is further reinforced by the presence of prebiotic components—including chitin and non-starch polysaccharides—which promote beneficial gut microbiota, improve gastrointestinal health, and may aid in the prevention of metabolic disorders [15]. In addition, recent studies have shown that wild-growing mushrooms such as *Leccinum scabrum* accumulate significant quantities of protocatechuic, vanillic, syringic, cinnamic, and hydroxybenzoic acids, as well as flavonoids including catechin, kaempferol, and rutin, further supporting their role as a source of bioactive compounds with antioxidant potential [19].

Although the biological properties of these compounds are well established, it is important to consider their stability under food processing conditions. Thermal treatments such as drying, cooking, or baking may lead to partial degradation or transformation of phenolic compounds—particularly flavonoids and phenolic acids—resulting in reduced or altered antioxidant activity [20,21]. Likewise, β-glucans may undergo slight physicochemical modifications when exposed to elevated temperatures; however, numerous studies indicate that under conventional processing conditions (typically below ~120 °C), they retain most of their structural integrity and functional properties [22]. Notably, studies investigating mushroom drying have shown that more than 50% of the initial antioxidant activity can be preserved following thermal processing [23,24]. These findings support the practical application of mushroom-derived bioactive compounds in functional foods, provided that processing parameters are appropriately optimised.

Nonetheless, wild mushrooms can also pose health risks. In addition to the risk of misidentification with toxic species, mushrooms are known bioaccumulators of environmental contaminants, including heavy metals such as cadmium (Cd), lead (Pb), mercury (Hg), and arsenic (As) [25,26,27,28]. Elevated concentrations of these toxic elements have been reported in edible species such as *Boletus edulis*, *Cantharellus cibarius*, and *Macrolepiota procera*, particularly in regions exposed to industrial or traffic-related pollution [29,30]. Chronic intake of such contaminated mushrooms—especially from areas with high anthropogenic pressure—can pose toxicological hazards, as Cd and Pb are both carcinogenic and neurotoxic and may lead to renal dysfunction, neurological damage, and cardiovascular complications [31], and recent research confirms that *I. badia* can bioaccumulate significant concentrations of various elements (including non-essential and toxic) that reflect those present in the substrate soil. Moreover, such accumulation may be associated with phenolic content in mushroom fruiting bodies, and specimens of *I. badia* collected from polluted sites may exhibit altered antioxidative capacity compared to those from unpolluted areas. At the same time, the flavonoid content in *I. badia* was found to be consistently low and remained unaffected by the concentration of bioaccumulated elements [32].

Polyphenols have been identified as key agents in the chelation and detoxification of heavy metals in fungi [33,34,35]. Through their hydroxyl and carbonyl functional groups, these compounds can form stable complexes with toxic metals such as Cd, Pb, and Ni, thereby reducing their bioavailability and toxicity. Concurrently, these complexes maintain the antioxidant role of phenolics and enhance the organism’s defence mechanisms under environmental stress.

Heavy metal bioaccumulation in mushroom fruiting bodies is influenced by both intrinsic and extrinsic factors. Species-specific metabolic pathways, morphology (e.g., cap and stipe structure), developmental stage, and mycelial age all affect elemental uptake. Environmental variables such as soil type, pH, organic matter content, moisture, climate, and anthropogenic impact further modulate accumulation patterns [36,37,38].

In light of this complexity, detailed investigations of selected mushroom species across different geographic regions are essential to assess both their nutritional potential and food safety. For instance, *Boletus edulis* harvested in the Polish Carpathians may exhibit a markedly different phenolic and β-glucan profile than the same species in Mediterranean environments [30], and may bioaccumulate different levels of heavy metals depending on local pollution [25,29]. Similarly, *Morchella* spp. (North America) and *Lentinula edodes* (Asia) display distinct metabolic characteristics which influence their application in food and health sciences [15,39]. Only through comprehensive characterisation of regional fungal populations can we fully understand their value in functional diets and nutraceutical applications [1].

Given this context, the present study focused on three wild edible mushroom species of high nutritional relevance in Central and Eastern Europe: *Boletus edulis*, *Imleria badia*, and *Leccinum scabrum*. Fruiting bodies were collected from two forested regions in north-western Poland, characterised by differing degrees of anthropogenic influence. The aims of the study were: (1) to analyse and compare the profiles of bioactive compounds (including polysaccharides, carotenoids, organic and phenolic acids) in relation to geographical origin; (2) to evaluate the antioxidant activity of the selected species and identify factors driving this activity; (3) to investigate the relationship between soil chemistry and mushroom biochemical profiles; and (4) to quantify the levels of Cd, Pb, and Ni in fruiting bodies and assess the associated dietary risks.

## 2. Results and Discussion

### 2.1. Soil Properties

The study, conducted in the forested physical-geographical regions of Uznam and Wolin as well as the Ińsko Lake District (north-western Poland), revealed that *Boletus edulis*, *Imleria badia*, and *Leccinum scabrum* occur predominantly in sandy, acidic, and nutrient-poor soils (Table 1), classified as Arenosols [40]. In the Uznam–Wolin region, soils beneath *B. edulis* exhibited significantly lower organic matter content and electrical conductivity (salinity), as well as higher pH values (measured in KCl), in comparison with soils supporting *I. badia* and *L. scabrum* (Table 1).

The concentrations of cadmium (Cd), nickel (Ni), and lead (Pb) were also found to be higher in soils collected beneath *I. badia* and *L. scabrum* in the Uznam–Wolin region. Notably, the two study areas differ markedly in terms of anthropogenic influence. The Ińsko Lake District remains relatively undisturbed by human activity, whereas the Uznam and Wolin area forms part of a coastal tourist zone traversed by a major thoroughfare with intense vehicular traffic. Combustion of vehicle fuel has been identified as a major contributor to elevated Cd, Pb, and Ni levels in forest soils located outside industrialised zones [26].

**Figure 1:**
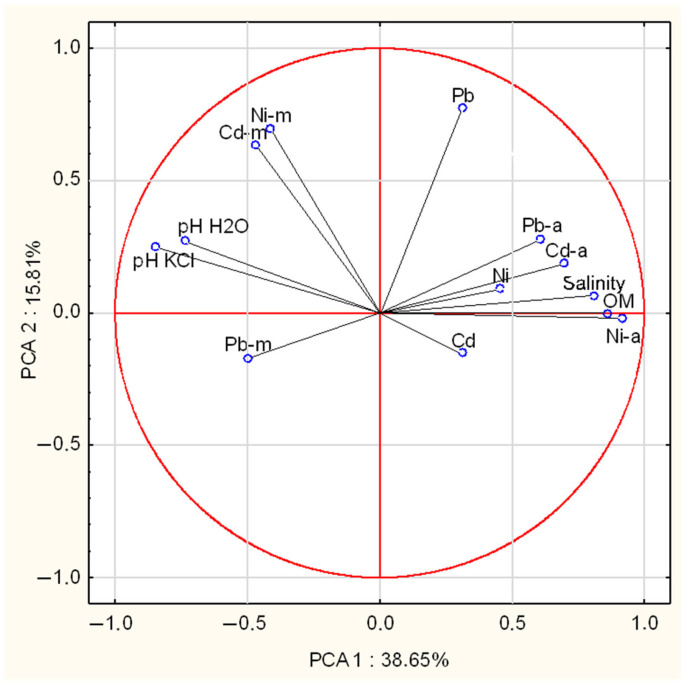
The principal component analysis (PCA) for soil and mushroom chemical composition (Cd-m—mushrooms, Cd-a—available, Cd—total, OM—organic matter).

**Figure 2:**
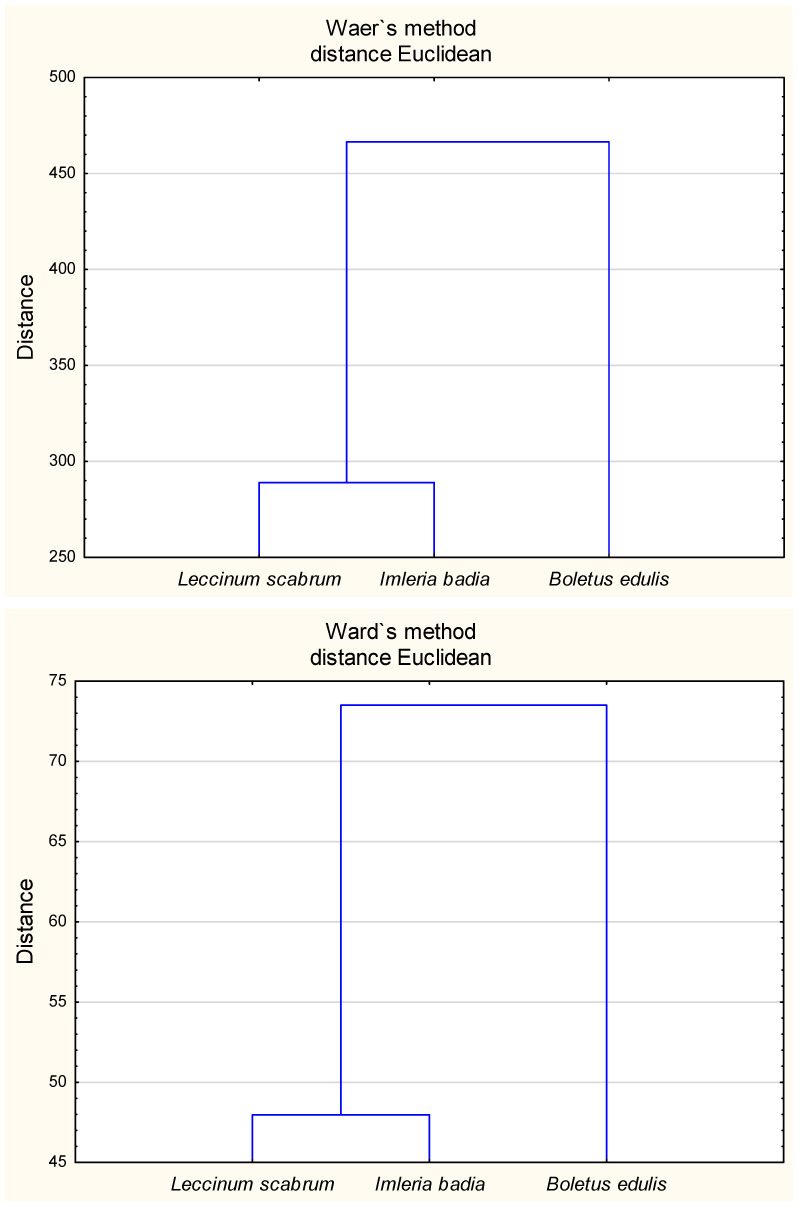
Ward’s cluster analysis for heavy metals content in soils (**top**) and in the mushrooms (**bottom**) where the mushrooms grew (*L. scabrum*, *I. badia*, *B. edulis*).

Principal component analysis (PCA) revealed that both available and total fractions of Cd, Ni, and Pb in the soil were influenced by organic matter content and pH (Figure 1). When all chemical parameters were taken into account, Ward’s hierarchical cluster analysis demonstrated that soils beneath *I. badia* and *L. scabrum* were closely related, yet distinctly different from those supporting *B. edulis* (Figure 2).

### 2.2. Bioactive Compounds in Boletus edulis, Imleria badia, and Leccinum scabrum

#### 2.2.1. Polysaccharides, Carotenes, and Ascorbic Acid

The analysis of bioactive compounds revealed marked interspecific differences among the mushroom species studied (Table 2). *Boletus edulis* exhibited the highest concentrations of the two primary cell wall polysaccharides—β-glucan and α-glucan (46.6 g/100 g DW and 3.93 g/100 g DW, respectively)—as well as lycopene (1.36 mg/100 g DW), while presenting the lowest level of L-ascorbic acid (20.2 mg/100 g DW). By contrast, *Imleria badia* contained the highest concentration of L-ascorbic acid (43.35 mg/100 g DW) and the lowest level of α-glucans (3.31 g/100 g DW). *Leccinum scabrum* recorded the lowest β-glucan (25.2 g/100 g DW) and lycopene contents (0.65 mg/100 g DW).

Across all species, β-glucans consistently constituted the predominant fraction among the extracted polysaccharides, followed by chitin and α-glucans. However, Santoyo et al. [41] reported a different polysaccharide hierarchy in *B. edulis*, with chitin exceeding both β- and α-glucans. Furthermore, Valentão et al. [42] did not detect ascorbic acid in fruiting bodies of *B. edulis* collected in Portugal. No statistically significant interspecific differences were observed in the content of chitin and β-carotene (Table 2). The α-glucan levels measured in the present study were consistent with values previously reported for these species in north-eastern Poland. In contrast, the β-glucan content recorded in *B. edulis* and *I. badia* was notably higher and among the highest values reported for edible mushrooms [43], reflecting their superior capacity for nutrient accumulation and their value as functional food components.

Elevated glucan concentrations enhance both the nutritional and therapeutic potential of mushrooms. Dietary supplements derived from mushrooms have been linked to a broad spectrum of health benefits [44]. β-glucans, as soluble dietary fibres, are known to lower blood cholesterol and regulate glycaemic levels. Their consumption may support cardiovascular function and reduce the risk of metabolic disorders such as type 2 diabetes and obesity [10,45]. Additionally, β-glucans exhibit immunomodulatory effects by stimulating macrophages and strengthening host defence mechanisms [46].

Conversely, α-glucans serve primarily as energy storage compounds and are readily assimilated by the human body. The relatively stable α-glucan levels observed across species suggest their potential as a consistent dietary energy source, particularly in the formulation of functional food products. Alongside α- and β-glucans, chitin represents a major structural component of fungal cell walls. This insoluble polysaccharide displays prebiotic activity, promoting the growth of beneficial intestinal microbiota, enhancing peristalsis, and supporting gastrointestinal health [47]. Moreover, chitin may contribute to increased satiety, which is of particular interest in the context of weight management. In combination with β-glucans, it constitutes a valuable source of dietary fibre with broad-spectrum health-promoting properties.

All three mushroom species exhibited relatively low levels of β-carotene and lycopene (Table 2). These findings are consistent with those of Kalač [48], who noted the presence of carotenoids in *B. edulis* as precursors of retinol. Jaworska et al. [49] further demonstrated that fresh fruiting bodies of *B. edulis* contain higher levels of β-carotene and lycopene than those subjected to thermal processing.

The results of the present study also indicate that environmental conditions significantly affected the concentrations of the analysed bioactive compounds (Table 2). This was particularly evident in the variation in chitin content in *L. scabrum* and in the levels of L-ascorbic acid across all species. These findings underscore the role of edaphic and microclimatic factors in modulating mushroom biochemical composition and point to the possibility of habitat selection as a strategy for optimising the nutritional value of wild mushrooms.

#### 2.2.2. Organic Acids in Mushroom

Table 3 presents a comprehensive analysis of the organic acid profiles in the fruiting bodies of three mushroom species: *Boletus edulis*, *Imleria badia*, and *Leccinum scabrum*. The results reveal distinct interspecific differences in metabolic composition. Most of the identified compounds belong to the most commonly occurring phenolic acids found in macrofungi, including gallic acid, p-coumaric acid, vanillic acid, ferulic acid, and caffeic acid, whereas chlorogenic acid was less frequently detected [50,51,52,53]. In *B. edulis*, p-coumaric acid predominated, accompanied by a substantial concentration of oxalic acid. In contrast, citric acid was the dominant compound in *I. badia*, while *L. scabrum* was characterised by high levels of catechin (Table 3). Notably, quinic acid was detected exclusively in *L. scabrum*, underscoring its distinctive metabolic profile, likely associated with species-specific enzymatic pathways.

*B. edulis* exhibited the highest average concentration of oxalic acid (45.00 mg/100 g), consistent with findings by Royse et al. [54], which may indicate its prominent role in nutrient cycling within forest ecosystems. *I. badia* displayed intermediate levels of oxalic acid, while *L. scabrum* showed the lowest values, further highlighting metabolic divergence among species. At elevated concentrations, oxalic acid is classified as an anti-nutritional compound due to its ability to chelate essential minerals such as calcium, magnesium, and iron, thereby limiting their bioavailability and intestinal absorption [55,56]. Chronic intake of high amounts of soluble oxalates has been associated with an increased risk of calcium oxalate kidney stone formation and, in rare cases, may contribute to oxalate nephropathy (hyperoxaluria) in susceptible individuals [57,58].

The highest mean level of fumaric acid (13.67 mg/100 g) was observed in *I. badia*, suggesting a potentially more active tricarboxylic acid (Krebs) cycle in this species compared with *B. edulis* and *L. scabrum* [39].

Citric acid content also varied substantially among species. *I. badia* accumulated the highest concentration (128.34 mg/100 g), indicating a greater biosynthetic capacity or storage efficiency for this compound. In contrast, *B. edulis* displayed the lowest citric acid levels, potentially reflecting reliance on alternative metabolic pathways [12]. *L. scabrum* exhibited intermediate values, approximately ten times lower than those observed in *I. badia*.

Succinic acid, an important intermediate in primary metabolism, was detected in *B. edulis* (2.18 mg/100 g) and *L. scabrum* (0.70 mg/100 g), but was absent in *I. badia*. These findings align with previous reports indicating low succinic acid content in *B. edulis* and related species [42].

Among the phenolic acids with known antioxidant activity, such as ferulic, chlorogenic, and caffeic acids [59], *B. edulis* generally exhibited the highest concentrations, while in *I. badia*, only chlorogenic acid was detected. p-Coumaric acid, a hydroxycinnamic acid derivative, was present solely in *B. edulis* and in relatively high amounts. This is noteworthy, given the broad biological activity attributed to p-coumaric acid, including antioxidant [15,60], antimutagenic [61], anticancer [62], and antibacterial effects [63].

Gallic acid, a potent antioxidant [15,64], was detected in all three species, with the highest concentrations observed in *L. scabrum* and only trace amounts in *I. badia*. Other hydroxybenzoic acid derivatives, such as salicylic and vanillic acid, were not uniformly present across species. Salicylic acid was absent in *B. edulis*, while vanillic acid was not detected in *I. badia*.

Salicylic acid plays a key role in plant and fungal stress responses by inducing the synthesis of defence-related metabolites, including phenolic compounds and antioxidants [65,66].

Notably, *I. badia* lacked or contained only trace amounts of six of the organic acids analysed. Nonetheless, its elevated levels of citric and fumaric acids suggest it may serve as a natural source of compounds with energy-enhancing and metabolic regulatory potential. Conversely, the considerable accumulation of oxalic acid in *B. edulis* aligns with its culinary applications, where acidic notes are often desirable. The exclusive presence of quinic acid in *L. scabrum* indicates species-specific biosynthetic pathways and warrants further biochemical investigation. Additionally, *L. scabrum* contained significantly higher levels of catechins than the other species (Table 3). These concentrations exceeded those reported for 26 other mushroom species (1.33 ± 0.31 to 20.50 ± 1.26 µg/g; [67], suggesting a particularly high antioxidant potential and enhanced bioavailability of functional metabolites [68].

In addition to catechin, *L. scabrum* demonstrated the presence of several other phenolic compounds contributing to its antioxidant profile [19]. Among the hydroxybenzoic acids, protocatechuic acid (4.21 mg/g DW) and vanillic acid (0.94 mg/g DW) were present in considerable quantities, alongside moderate levels of syringic (0.52), 2,5-dihydroxybenzoic (0.61), and 4-hydroxybenzoic acids (0.61). Among the hydroxycinnamic acids, a particularly high content of t-cinnamic acid was noted (12.57 mg/g DW), indicating potential for significant radical scavenging activity. The total quantified phenolics in *L. scabrum* reached 22.90 mg/g DW. In the flavonoid group, kaempferol (0.41), catechin (1.61), apigenin (0.39), and rutin (0.26) were detected, while luteonin, naringinin, quercetin, and vitexin were below the detection threshold. This diverse spectrum of bioactive compounds reinforces the chemotaxonomic distinctiveness of *L. scabrum* and supports its potential as a functional food ingredient with health-promoting properties.

While the primary focus of the study was on interspecific metabolic variation, the impact of site-specific environmental conditions—particularly soil composition and microclimate—was also evident. The concentrations of organic acids in mushroom fruiting bodies are shaped by both intrinsic species-specific metabolic traits and extrinsic habitat conditions [69].

Sampling location significantly influenced the levels of certain organic acids (Table 3). For example, *B. edulis* collected from Uznam–Wolin exhibited higher oxalic acid content (52.94 mg/100 g) than samples from the Ińsko Lake District (37.06 mg/100 g). Similarly, *I. badia* from the Ińsko Lake District showed higher oxalic acid levels (42.78 mg/100 g) than those from Uznam–Wolin (24.67 mg/100 g), likely due to differences in soil nutrient availability or microclimatic conditions.

Fumaric acid content in *I. badia* also showed substantial regional variation, with higher values in Uznam–Wolin (21.56 mg/100 g) compared to the Ińsko Lake District (5.78 mg/100 g). In contrast, citric acid content was slightly greater in *I. badia* from the Ińsko Lake District (128.34 mg/100 g) than from Uznam–Wolin (112.26 mg/100 g). Meanwhile, *L. scabrum* maintained relatively stable citric acid levels across both locations, suggesting a greater resilience to environmental fluctuations.

Regional differences in phenolic acid concentrations were also observed, though to a lesser extent. *B. edulis* from Uznam–Wolin exhibited higher chlorogenic acid levels than samples from the Ińsko Lake District. A similar trend was observed for gallic acid in *L. scabrum*, whereas catechin concentrations in *L. scabrum* were higher in specimens from the Ińsko Lake District. These findings are likely linked to site-specific soil and climate conditions influencing polyphenol synthesis, consistent with patterns observed in plants [70,71,72].

The phenolic profile of *B. edulis* has also been characterised in detail by Fogarasi et al. [73], who identified 17 individual polyphenolic compounds. Among them, protocatechuic acid 4-O-glucoside (1735.4 µg/g), syringic acid (934.2 µg/g), and 2,4-dihydroxybenzoic acid (590.7 µg/g) were the most abundant. The presence of both hydroxybenzoic acids and a variety of flavonoid glycosides—including quercetin derivatives, catechin (122.5 µg/g), and epicatechin (74.1 µg/g)—demonstrates the chemical complexity of *B. edulis* beyond the standard organic acid profile. The total content of quantified polyphenols reached 4632.4 µg/g, supporting its high antioxidant potential. Notably, gallic acid was also present (371.5 µg/g), aligning with the results of the present study. These data suggest that *B. edulis* can serve as a valuable source of diverse phenolic compounds with confirmed bioactivity.

Overall, these results underscore the complex interplay between intrinsic metabolic strategies and environmental influences in determining mushroom chemical composition. While species-specific factors are primary determinants of metabolite profiles, the contribution of regional environmental conditions—such as soil characteristics and microclimate—must not be overlooked. This nuanced understanding of organic and phenolic acid distribution has important implications for the use of wild mushrooms in functional food and nutraceutical applications.

A summary of the bioactive compounds found in *B. edulis*, *I. badia*, and *L. scabrum* should take into account that, in the context of practical applications of bioactive compounds, their bioavailability—that is, the capacity to be absorbed and utilised by the human body—is equally important. In mushrooms, particular attention has been given to polysaccharides (especially β-glucans) and phenolic compounds, which are considered key contributors to their health-promoting potential [74,75]. However, the presence of these compounds within the natural mushroom matrix—characterised by the complex structure of cell walls composed of chitin and β-glucans, and their chemical associations with other constituents—may limit their bioaccessibility [76].

Due to their complex and partially insoluble structure, fungal polysaccharides often require appropriate processing—such as aqueous extraction, enzymatic treatment, or fermentation—to be efficiently released from the mushroom matrix and absorbed in the gastrointestinal tract. Moreover, their biological activity is closely linked to structural features such as branching degree, molecular weight, and helical conformation, all of which influence the immunomodulatory properties of β-glucans [76]. Similarly, phenolic compounds are frequently bound to proteins or polysaccharides, which can hinder their bioavailability. Studies have shown that processing methods such as grinding, thermal treatment, or fermentation can significantly enhance the bioavailability of both phenolics and polysaccharides [77,78].

Thus, not only the quantitative and qualitative composition of bioactive compounds in mushrooms but also their localisation and molecular form within the mushroom matrix play a critical role in determining their actual nutraceutical value. This perspective opens new possibilities for the development of mushroom-based functional foods by considering both their intrinsic composition and the optimisation of processing techniques aimed at enhancing bioavailability.

### 2.3. Antioxidant Activity of Boletus edulis, Imleria badia, and Leccinum scabrum

The results indicated that both mushroom species and sampling location significantly influenced not only the biochemical composition but also the antioxidant activity of bioactive compounds (Table 4). The outcomes of the DPPH, ABTS, and FRAP assays—each applied to the same mushroom samples—differed at times considerably. Similar inconsistencies have been reported for other species such as *Ganoderma lucidum*, *Lentinus edodes*, and *Agaricus bisporus* [79]. These variations are likely attributable to the diverse antioxidant compounds present in the samples, which exhibit differing affinities and reaction mechanisms with the radical systems employed in each assay.

Nevertheless, irrespective of the assay applied, statistically significant interspecific differences in antioxidant potential were observed. Among the species tested, *Leccinum scabrum* demonstrated the highest antioxidant values in both the ABTS and FRAP assays, yet exhibited the lowest DPPH radical scavenging capacity. In contrast, *Imleria badia* showed the highest activity in the DPPH assay, but only moderate results in the ABTS and FRAP tests. *Boletus edulis*, by comparison, displayed the weakest reducing power in the FRAP assay and the lowest ABTS radical scavenging capacity. Interestingly, strong antioxidant properties of *B. edulis* using the same set of assays (DPPH, ABTS, FRAP) have been previously reported in specimens from Brazil [80].

### 2.4. Heavy Metals in Mushrooms

Despite being collected from habitats with similar environmental conditions, the examined mushroom species exhibited varying levels of nickel (Ni), cadmium (Cd), and lead (Pb) accumulation, with *Boletus edulis* generally containing the highest concentrations of these elements (Table 5).

**Figure 3:**
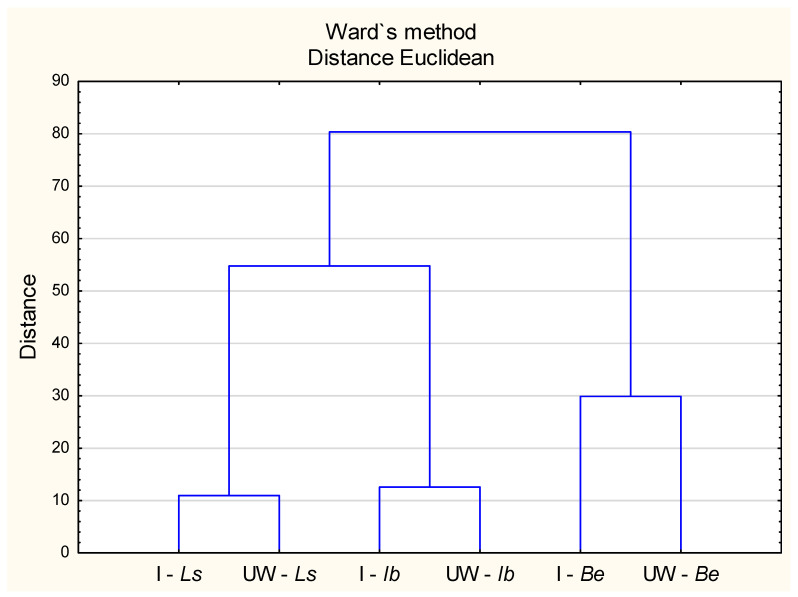
Ward’s cluster analysis of heavy metals content in mushrooms (*L. scabrum*, *I. badia*, *B. edulis*) with respect to their location. Explanations: I—Ls: physical-geographical region Ińsko Lake District—*L. scabrum*; UW—Ls: physical-geographical region of Uznam and Wolin—*L. scabrum*.

Elemental uptake by the analysed mushrooms was positively correlated only with soil pH; no statistically significant relationships were observed with organic matter content or with the total and available fractions of Cd, Ni, and Pb in soil (Figure 1). The relationship between soil pH and metal uptake appears to be species-dependent. For example, Pb uptake was found to be negatively correlated with pH in *Coprinus comatus*, while a positive correlation was observed for Ni in *Volvariella gloiocephalus* [37]. In contrast, Malinowska et al. [81] did not report any consistent influence of soil pH on heavy metal uptake in fungi. Our findings support the view that, in addition to physiological factors, the environmental context—particularly soil characteristics—plays a decisive role in determining the elemental composition of mushroom fruiting bodies (Figure 2 and Figure 3; Table 5).

The concentrations of Cd, Pb, and Ni in the mushrooms analysed in this study were within the ranges typically reported for wild-growing fungal species in Poland and elsewhere in Europe [82,83,84,85,86,87].

Mushrooms are known for their ability to absorb and bioaccumulate mineral elements, often at levels exceeding those found in surrounding soil [27,88]. However, this capacity varies considerably between species and is highly dependent on site-specific soil conditions. In the present study, none of the analysed species showed evidence of Pb or Ni bioaccumulation (Table 6). The lack of Pb accumulation is in agreement with earlier studies [87,89,90,91], although some fungi have been shown to accumulate lead under specific environmental conditions [92,93]. Similarly, Ni bioaccumulation is typically observed only in soils with abnormally high nickel concentrations [72,87].

Among the species examined, *Boletus edulis* was the only one to consistently bioaccumulate cadmium across both study locations (Table 6), a finding consistent with data from other forest ecosystems [87,92,94]. Similar Cd levels have been reported in *B. edulis* fruiting bodies collected in Croatia [95]. *Leccinum scabrum* also demonstrated a tendency to bioaccumulate Cd, but only in specimens collected from the Ińsko Lake District. These results illustrate the influence of both species-specific uptake mechanisms and environmental factors—such as soil chemistry, elemental bioavailability, and possible antagonistic or synergistic interactions—on metal accumulation patterns. This pattern is further confirmed by findings from Gąsecka et al. [32], who analysed the content of chemical elements in *Imleria badia* and soils from both unpolluted and polluted sites. Their data demonstrate a clear increase in cadmium, lead, and zinc content in both soils and mushroom tissues collected from contaminated areas. Notably, *I. badia* fruiting bodies from polluted sites exhibited over three-fold higher Cd levels (0.60–0.86 mg·kg^−1^ dm) compared to those from unpolluted soils (0.20–0.26 mg·kg^−1^ dm). A similar trend was observed for Pb, which increased from 0.15–0.23 mg·kg^−1^ dm in mushrooms from clean areas to 2.09 mg·kg^−1^ dm, despite relatively moderate Pb levels in soil. In the case of nickel, a marked difference was also found: while concentrations in mushrooms from unpolluted sites ranged between 0.16 and 0.22 mg·kg^−1^ dm, specimens from polluted areas accumulated significantly more Ni—up to 1.16 ± 0.17 mg·kg^−1^ dm—indicating elevated environmental availability and potential bioaccumulation under anthropogenic pressure. These site-dependent differences reinforce the importance of monitoring local pollution when assessing edible mushroom safety.

All measured concentrations of Cd, Pb, and Ni in the examined mushrooms were below the maximum allowable levels for human consumption as defined by EU regulations [96], indicating that the samples are safe for dietary use. Nevertheless, regular and excessive consumption of *B. edulis* may result in exceeding the tolerable weekly intake levels, particularly for cadmium and nickel [97,98,99]. These findings underscore the importance of moderate consumption of wild mushrooms—especially those species known to accumulate toxic elements—as long-term intake may pose potential health risks [25]. However, it is important to note that the actual dietary exposure to toxic elements may be lower than estimated from total concentrations in raw mushrooms. Several studies have demonstrated that cooking and gastrointestinal digestion substantially reduce the bioaccessibility of heavy metals. For instance, Chiocchetti et al. [100] reported that thermal processing and in vitro digestion reduced toxic element levels in mushrooms, among others in *B. edulis*, where the bioaccessibility of Cd did not exceed 40%, while that of Pb was below the detection limit of the applied method. Similar findings were observed by Sun et al. [101], who showed that cooking significantly reduced cadmium availability in *Agaricus blazei*. These results suggest that food safety assessments should consider not only total metal content but also processing conditions and bioavailability in order to avoid overestimating health risks.

The high affinity of mushrooms for heavy metals can be partly attributed to the presence of chitin in their cell walls. Chitin and its modified derivatives exhibit a strong biosorptive capacity for divalent metal ions, including Cd^2+^, Pb^2+^, and Zn^2+^, with adsorption efficiencies exceeding 100 mg/g under laboratory conditions [102]. Various processing techniques—such as deacetylation (yielding chitosan), mechanical milling, and chemical or enzymatic modifications—significantly enhance the metal-binding capacity of chitin by exposing and activating functional groups (amine and hydroxyl) responsible for Cd^2+^ ion complexation [103].

In this context, chitin- or chitosan-rich extracts may serve as natural biosorbents not only for reducing cadmium bioaccessibility in mushrooms but also in other food matrices such as beverages, vegetable products, or animal-derived foods [104]. Furthermore, they represent renewable sorbents with promising efficacy in water purification systems [105]. The incorporation of such components could contribute to lowering free metal ion levels in final food products, offering an innovative strategy to enhance food safety.

### 2.5. Relationships Between the Chemical Composition of Mushroom Fruiting Bodies, Antioxidant Activity, Phenolic Compounds, and Heavy Metal Content

**Figure 4:**
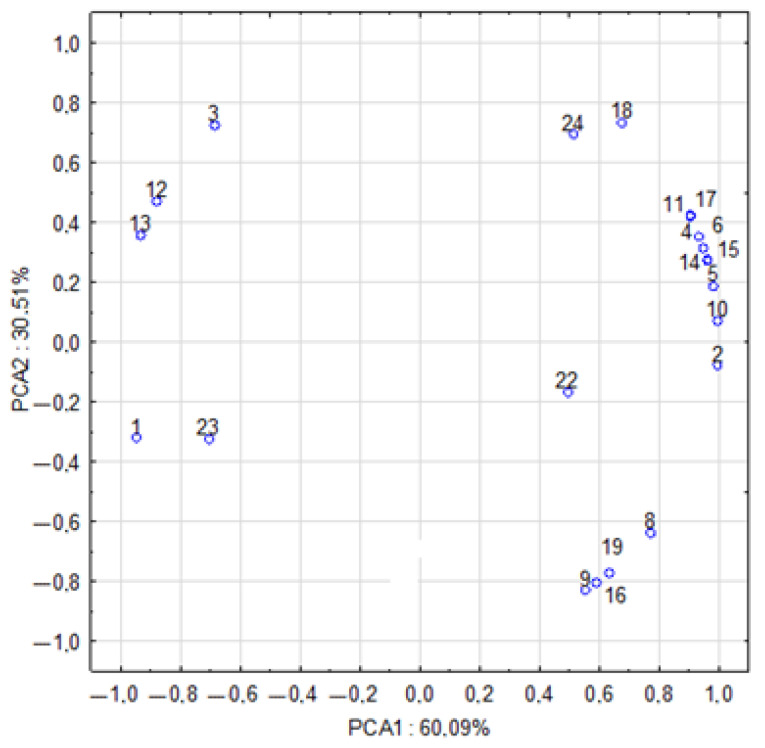
Principal component analysis (PCA) illustrating the relationships between the chemical composition of mushroom fruiting bodies, antioxidant activity, phenolic compounds, and heavy metal content. Explanations: 1—chitin; 2—β-glucan; 3—α-glucan; 4—DPPH·; 5—ABTS·+; 6—FRAP; 8—L-ascorbic acid; 9—β-carotene; 10—lycopene; 11—quercetin; 12—catechin; 13—caffeic acid; 14—chlorogenic acid; 15—ferulic acid; 16—galic acid; 17—salicylic; 18—vanillic; 19—p-coumaric acid; 22—Ni; 23—Cd; 24—Pb.

Principal component analysis (PCA) of the chemical composition and antioxidant activity of the mushrooms revealed several key relationships among the variables assessed (Figure 4). A strong positive correlation was observed between the antioxidant assays DPPH·, ABTS·+, and FRAP, with the closest association found between DPPH· and FRAP, despite the assays relying on different reaction mechanisms. Similar correlations have been reported in studies on wild edible mushrooms from Bulgaria [106] and phenolic extracts from *Malus* wild species [107]. In contrast, weaker correlations between DPPH and FRAP were noted in lignin [108] and vegetable juice studies [109], suggesting that sample origin and matrix complexity can significantly influence assay concordance. These findings support the reliability of the selected methods in evaluating antioxidant capacity in mushrooms.

Among the tested compounds, phenolic acids—particularly chlorogenic, ferulic, salicylic, and quercetin—demonstrated strong correlations with antioxidant activity in all three assays. This underscores their critical role in the antioxidant potential of mushrooms, akin to their established roles in plants [110]. Although fungal metabolic pathways more closely resemble those of animals, these phenolic compounds appear essential to sporocarp protection and are likely synthesised through diverse biosynthetic routes [59].

By contrast, structural components such as chitin and α-glucans showed no direct relationship with antioxidant capacity, suggesting that their primary functions are structural or mechanical rather than chemical. This observation aligns with broader findings on the biological roles of polysaccharides and highlights their significance in food technology and biopreparation contexts. Notably, α-glucan derived from *Hericium erinaceus* has been shown to promote the growth of beneficial gut bacteria (*Lactobacillus*, *Bifidobacterium*) while inhibiting pathogenic *Fusobacterium* species [14].

Regarding heavy metals, cadmium exhibited a negative correlation with both antioxidant activity and certain phenolic compounds, indicating that its presence may inhibit the synthesis or stability of antioxidant molecules. Conversely, a strong positive correlation was observed between cadmium and chitin content, consistent with previous findings that chitin binds Cd via hydroxyl and acetylamino groups [111,112,113]. Nevertheless, the toxicological implications of heavy metal presence in mushrooms require further investigation, particularly with respect to environmental exposure and consumer safety. Based on in vitro data, it is assumed that the actual bioavailability of these elements after culinary processing and digestion is limited and unlikely to pose immediate health risks [100]. Importantly, experimental data confirm that culinary processing significantly reduces the bioaccessibility of cadmium from mushrooms. For instance, Sun et al. [101] showed that cooking *Agaricus blazei* decreased Cd bioaccessibility from ~77.8% in raw samples to ~50.7% after boiling and ~58.2% after microwaving. Such findings suggest that estimations of health risk based solely on total Cd content in raw mushrooms may overestimate actual dietary exposure under typical consumption conditions.

In our study, antioxidant activity was primarily associated with the profile of phenolic compounds and the presence of low-molecular-weight reductants. Species-specific antioxidant signatures were observed: *I. badia* showed the highest radical-scavenging capacity in the DPPH assay, while *L. scabrum* exhibited the strongest responses in ABTS and FRAP assays, which corresponded with its higher content of selected phenolics (e.g., catechin) and phenolic acids. In contrast, *B. edulis* was characterised by a greater proportion of polysaccharide fractions (β and αglucans), which—despite their well-documented physiological effects (e.g., immunomodulatory)—contribute minimally to the chemical assays employed here, which are dominated by low-molecular-weight antioxidants such as phenolics, ascorbic acid, and selected organic acids.

The relationship between antioxidant activity and heavy metal content appeared to be species- and environment-dependent. No clear linear trend was observed (e.g., higher metal content corresponding to higher or lower antioxidant activity). At least two opposing mechanisms may be involved: (i) chelation—free hydroxyl and carbonyl groups in phenolic compounds, as well as chitin/chitosan, may bind metal cations (e.g., Cd, Pb, Ni), thereby reducing their reactivity while simultaneously immobilising a portion of phenolics, which can impair their extraction and reactivity in in vitro assays; and (ii) stress response—exposure of fungal mycelium or fruiting bodies to environmental stressors, including heavy metals, may stimulate the biosynthesis of phenolic compounds, resulting in locally increased antioxidant capacity in samples collected from more contaminated sites. Consequently, these relationships may vary in direction and strength depending on species, metal form (soluble vs. bound), chemical matrix, and site history.

From a food safety perspective, it is important to note that antioxidant activity was primarily determined by the phenolic and ascorbic acid content, whereas heavy metal concentrations—although toxicologically relevant—did not directly enhance antioxidant potential. Notably, even in samples with elevated cadmium levels, the antioxidant activity could be attributed to the presence of reductive compounds rather than metals themselves. In practical terms, this suggests that the evaluation of functional value (phenolics, ascorbate, DPPH/ABTS/FRAP activity) and safety assessment (Cd, Pb, Hg, As) should be considered separately: the former supports the development of food applications, while the latter informs regulatory compliance and the selection of safe raw material sources.

In summary, the fruiting bodies of the studied mushroom species are rich in bioactive compounds and represent valuable sources of natural antioxidants. Their compositional diversity—which includes phenolics, carotenoids, organic acids, and polysaccharides—supports their potential use as functional ingredients in dietary supplements, biotechnology, and agriculture. However, the concurrent presence of heavy metals, especially cadmium, highlights the need for regular monitoring and site-specific assessment of wild mushrooms intended for human consumption to ensure food safety and public health. The results indicate that powders from *Boletus edulis*, *Imleria badia*, and *Leccinum scabrum* have significant potential for use in functional foods and clean-label applications. *B. edulis*, due to its umami profile and techno-functional properties, may serve as a natural flavour enhancer and texture agent in plant-based meat alternatives, baked goods, and instant mixes. *I. badia*, with high antioxidant activity, is suitable for applications requiring oxidative stability, such as wholegrain products, plant-based deli items, and emulsified fillings. *L. scabrum*, with its mild flavour and lighter colour, fits well in wheat bread, gluten-free blends, and powdered sauces where sensory neutrality is desirable. Application levels should be optimised through pilot testing, considering water-binding properties. Given the wild origin of the mushrooms, safety compliance (e.g., contaminants, metals, allergens) must be ensured. These powders can support the development of nutritionally enhanced, oxidatively stable, and clean-label food products.

## 3. Materials and Methods

### 3.1. Study Area and Sampling

Soil and mushroom samples were collected from two major forested regions in north-western Poland: (1) the northern coastal belt encompassing Uznam and Wolin, and (2) the southern area of the Ińsko Lake District. These regions differ markedly in terms of anthropogenic pressure. Uznam and Wolin are located within a popular coastal tourist zone intersected by a major roadway and subject to significant recreational use due to the presence of numerous holiday resorts. In contrast, the Ińsko Lake District remains largely unaffected by direct human activity and is characterised by low levels of urbanisation.

The regions also differ climatically. Uznam and Wolin experience a milder climate, with more sunny and warm days and fewer precipitation events [114]. Mean annual precipitation is approximately 550 mm, and average annual air temperature ranges from 8.0 to 8.5 °C, reaching approximately 9.0 °C in the autumn [115]. By contrast, the Ińsko Lake District experiences a harsher climate, with more frequent rainfall, fewer warm days, and a higher incidence of frost. Annual precipitation ranges from 625 to 650 mm, with an average annual temperature of 7.5 °C and approximately 8.0 °C in autumn [115].

Soils in both regions are sandy and acidic, enriched with organic matter, and variable in macronutrient content. The substrate for *B. edulis* was richer in available potassium (K), magnesium (Mg), and phosphorus (P), whereas soils where *I. badia* and *L. scabrum* were found contained lower levels of available K and Mg [116,117].

The study focused on three widely foraged wild edible mushroom species: *Boletus edulis*, *Imleria badia*, and *Leccinum scabrum*. Composite samples of each species and corresponding soil samples were collected from both geographical regions. From each location, four to six composite samples per species were obtained. Each composite consisted of five fruiting bodies. In total, 10 composite samples (50 fruiting bodies) of *B. edulis*, 10 (50 fruiting bodies) of *I. badia*, and 8 (40 fruiting bodies) of *L. scabrum* were prepared, yielding 28 mushroom samples for analysis. The mushrooms were cleaned, dried at 35–38 °C for 48 h in an electric dryer, and ground into a fine powder without separating caps from stipes. Taxonomic identification was carried out using standard macroscopic methods, following Knudsen and Vesterholt [118], and verified using the Index Fungorum database (accessed on 20 May 2025; http://www.indexfungorum.org/).

### 3.2. Elemental Analysis

#### 3.2.1. Soil

Available forms of Cd, Ni, and Pb in the soil were determined by extraction with 0.5 mol·dm^−3^ HCl. Total concentrations of these elements were measured after mineralisation in concentrated HNO_3_ and HClO_4_ (1:1 ratio) using flame atomic absorption spectroscopy (iCE 3000 Series, Thermo Fisher Scientific, Waltham, MA, USA). Analytical accuracy and precision were assessed using certified reference material CRM036–050 Loamy Sand 4 (Resource Technology Corporation, State College, PA, USA). Recovery rates ranged between 90% and 95%. Values represent means of three independent measurements. Working standards were prepared from Merck (Merck KGaA, Darmstadt, Germany) solutions (1000 mg·dm^−3^).

#### 3.2.2. Mushrooms

Elemental concentrations of Cd, Ni, and Pb in mushroom samples were determined following wet digestion with H_2_SO_4_ and HClO_4_ (3:1 ratio). Measurements were carried out using the same atomic absorption system (iCE 3000 Series). The efficiency of the process was validated with 90–93% success using certified reference materials, namely, tea leaves (INCT-TL-1) and a mixture of Polish herbs (INCT-MPH-2), both produced by the Institute of Nuclear Chemistry and Technology, Warsaw, Poland. All analyses were performed in triplicate.

### 3.3. Bioactive Compounds and Health-Promoting Properties

#### 3.3.1. Determination of Bioactive Compounds

β-carotene and lycopene were analysed spectrophotometrically using extracts prepared with a 4:6 (*v*/*v*) mixture of acetone and hexane and subjected to sonication, following the method of Barros et al. [119]. A standard curve was constructed using gallic acid, and absorbance was measured at 700 nm. Results were expressed as mg gallic acid equivalents (GAE) per gram of dry weight.

Chitin content was determined as glucosamine following hydrolysis with 6N HCl and a subsequent colorimetric reaction, as described by Ride and Drysdale [120]. Glucans were measured in quadruplicate using a commercial assay kit (Megazyme Ltd., Bray, Ireland) and the manufacturer’s protocol. All enzymes were sourced from Megazyme Ltd.

L-ascorbic acid and nitrate levels were quantified using an RQflex 10 reflectometer (Merck, Darmstadt, Germany) according to the method described by Mijowska et al. [121]. All determinations were carried out in triplicate.

#### 3.3.2. Antioxidant Assays

To evaluate antioxidant capacity, mushroom extracts were obtained by maceration in 70% ethanol at 20 °C for 60 min under constant stirring. After centrifugation (4000 rpm, 10 min), the supernatant was filtered under reduced pressure through a 1.2 μm cellulose membrane and concentrated at 40 °C using a vacuum evaporator. The aqueous residue was diluted with distilled water.

Antioxidant activity was assessed using three methods: ABTS·+ (2,2′-azino-bis(3-ethylbenzothiazoline-6-sulphonic acid)) radical cation decolourisation [122], DPPH (1,1-diphenyl-2-picrylhydrazyl) radical scavenging [123], and FRAP (ferric reducing antioxidant power) assay [124]. Results were expressed as mmol Trolox equivalents (TE) per 100 g of extract. Measurements were conducted using a UV-2401 PC spectrophotometer (Shimadzu, Kyoto, Japan). All reagents were from Sigma-Aldrich (Steinheim, Germany), and all tests were performed in triplicate.

#### 3.3.3. Organic Acids

Organic acids were quantified using Ultra-Fast Liquid Chromatography (UFLC). A Shimadzu 20A series chromatograph (Shimadzu Corporation, Kyoto, Japan) was coupled with a photodiode array detector (PDA) set at 215 nm and 245 nm. Separation was achieved on a SphereClone C18 reverse-phase column (Phenomenex, Torrance, CA, USA; 5 μm, 250 mm × 4.6 mm i.d.) maintained at 35 °C. Elution was performed with 3.6 mM sulphuric acid at a flow rate of 0.8 mL/min. Peak areas were compared against external calibration curves constructed with commercial standards. Results were expressed in mg per 100 g dry weight.

### 3.4. Statistical Analysis

All statistical analyses were performed using Statistica 12.5 software (StatSoft Polska, Cracow, Poland). The normality of data distribution and homogeneity of variance were verified prior to applying one-way ANOVA with Tukey’s post hoc test. Statistical significance was established at *p* < 0.05. Multivariate analyses included principal component analysis (PCA), performed on automatically scaled data, and agglomerative hierarchical clustering using Ward’s method.

## 4. Conclusions

This study confirms that the profile of bioactive compounds in the fruiting bodies of *Boletus edulis*, *Imleria badia*, and *Leccinum scabrum* is shaped by both species-specific characteristics and environmental factors, including geographical location, soil properties, and anthropogenic pressure. Variability in the content of polysaccharides, carotenoids, organic acids, and phenolic compounds indicates that local microclimatic and edaphic conditions significantly influence the biosynthetic capacity of these fungi.

*B. edulis* exhibited the highest levels of β- and α-glucans as well as lycopene; *I. badia* was distinguished by elevated ascorbic and citric acid content; while *L. scabrum* contained the highest concentration of catechin and was the only species in which quinic acid was detected. The particularly high citric acid levels in *I. badia*, combined with its limited diversity of other organic acids, suggest a key role for this compound in shaping the antioxidant potential of the species.

PCA results revealed positive correlations between β-glucan, β-carotene, L-ascorbic acid, and various organic acids, indicating integrated metabolic networks contributing to antioxidant activity. A strong association was also confirmed between antioxidant performance (DPPH·, ABTS·+, FRAP) and the presence of phenolic compounds and polysaccharides. Among the species analysed, *I. badia* exhibited the greatest radical scavenging capacity (DPPH), while *L. scabrum* showed the strongest reducing power (ABTS, FRAP).

Mushroom chemical composition was found to be partly dependent on soil characteristics. Soils from the Uznam–Wolin region exhibited higher concentrations of heavy metals (Cd, Ni, Pb), and soil pH was identified as a key factor influencing their accumulation in fruiting bodies. No clear relationships were observed with soil organic matter content or with the availability of metal forms. Notably, secondary metabolites such as catechin and chitin showed a positive correlation with cadmium content, suggesting potential protective or detoxification-related roles.

Although heavy metals were not predominant in the biochemical profiles, their presence—particularly cadmium—warrants consideration when assessing the quality and safety of wild-harvested mushrooms. All metal concentrations measured were within permissible limits for human consumption. However, *B. edulis* consistently showed cadmium bioaccumulation across locations, and localised accumulation was also observed in *L. scabrum* from the Ińsko Lake District.

The high content of bioactive compounds, especially antioxidants, highlights the potential of *B. edulis*, *I. badia*, and *L. scabrum* as valuable sources of functional food ingredients, dietary supplements, and nutraceuticals. To enhance health benefits and reduce potential risks, dietary diversification through the combined consumption of different mushroom species—such as *I. badia* and *L. scabrum*—is recommended in order to optimise the intake of beneficial compounds while limiting heavy metal exposure.

## Figures and Tables

**Table 1:** Selected soil properties and exchangeable heavy metal content in soils from two physical-geographical regions for three mushroom species.

		Species of Mushroom		
	Location (Physical-Geographical Region)	*Boletus edulis*	*Imleria badia*	*Leccinum scabrum*
OM [%]	Uznam–-Wolin	5.06 a *	31.76 ab	64.83 b
	Ińsko Lake District	9.19 a	23.78 a	19.39 a
	mean	7.12 A	27.77 AB	42.11 B
pH in KCl	Uznam–Wolin	3.39 b	2.64 a	2.58 a
	Ińsko Lake District	3.17 b	2.98 ab	3.20 b
	mean	3.28 B	2.81 A	2.89 A
pH in H_2_O	Uznam–Wolin	3.72 ab	3.43 ab	3.32 a
	Ińsko Lake District	3.80 b	3.67 ab	3.53 ab
	mean	3.76 B	3.55 AB	3.42 A
Salinity [µS/cm]	Uznam–Wolin	91.94 a	271.35 c	214.83 bc
	Ińsko Lake District	132.80 ab	121.22 ab	129.05 ab
	mean	112.37 A	196.28 B	171.28 AB
Ni [mg/kg]	Uznam–Wolin	0.45 a	2.34 c	2.23 c
	Ińsko Lake District	1.10 b	1.40 b	1.37 b
	mean	0.77 A	1.87 B	1.80 B
Cd [mg/kg]	Uznam–Wolin	0.23 c	0.24 c	0.28 c
	Ińsko Lake District	0.01 a	0.06 ab	0.17 b
	mean	0.12 A	0.15 A	0.22 A
Pb [mg/kg]	Uznam–Wolin	10.08 ab	23.00 b	18.76 ab
	Ińsko Lake District	11.47 ab	11.69 ab	5.84 a
	mean	10.77 A	17.34 A	12.30 A

* Means marked with the same letters do not differ significantly at the significance level of *p* ≤ 0.05. Uppercase letters in the columns (vertical comparison) indicate comparisons between locations (Uznam–Wolin and Ińsko Lake District) for a given mushroom species. Uppercase letters in the last row of the table refer to comparisons of mean values between mushroom species. Lowercase letters indicate statistically significant (or non-significant) differences in the interaction between the examined factors.

**Table 2:** Bioactive compound content in fruit bodies of selected mushroom species by region.

		Species of Mushroom		
	Location (Physical-Geographical Region)	*Boletus edulis*	*Imleria badia*	*Leccinum scabrum*
Chitin (g/100 g)	Uznam–Wolin	22.9 bc *	20.9 ab	27.9 e
	Ińsko Lake District	24.7 d	23.6 cd	19.1 a
	mean	23.8 A	22.2 A	23.5 A
β-glucan (g/100 g)	Uznam–Wolin	48.6 f	30.3 c	23.7 a
	Ińsko Lake District	44.6 e	37.7 d	26.7 b
	mean	46.6 C	34.0 B	25.2 A
α-glucan (g/100 g)	Uznam–Wolin	3.72 bc	3.05 a	3.06 a
	Ińsko Lake District	4.13 c	3.56 b	4.11 c
	mean	3.93 B	3.31 A	3.59 AB
L-ascorbic acid (mg/100 g)	Uznam–Wolin	25.6 b	48.9 d	33.7 c
	Ińsko Lake District	14.8 a	37.8 c	24.5 b
	mean	20.2 A	43.35 C	29.1 B
β-carotene (mg/100 g)	Uznam–Wolin	2.78 cd	2.26 b	2.33 b
	Ińsko Lake District	1.98 a	2.82 d	2.65 c
	mean	2.38 A	2.54 A	2.49 A
Lycopene (mg/100 g)	Uznam–Wolin	1.43 d	1.02 c	0.74 bb
	Ińsko Lake District	1.28 d	0.76	0.55 a
	mean	1.36 C	0.89 B	0.65 A

* Means marked with the same letters do not differ significantly at the significance level of *p* ≤ 0.05. Uppercase letters in the columns (vertical comparison) indicate comparisons between locations (Uznam–Wolin and Ińsko Lake District) for a given mushroom species. Uppercase letters in the last row of the table refer to comparisons of mean values between mushroom species. Lowercase letters indicate statistically significant (or non-significant) differences in the interaction between the examined factors.

**Table 3:** Organic acids content in fruit bodies of selected mushroom species by region (µg/g = mg/100 g).

		Species of Mushroom		
(mg/100 g d.w.)	Location (Physical-Geographical Region)	*Boletus edulis*	*Imleria badia*	*Leccinum scabrum*
Quinic acid	Uznam–Wolin	n.d.	n.d.	1.04 b *
	Ińsko Lake District	n.d.	n.d.	0.55 a
	mean			1.67
Oxalic acid	Uznam–Wolin	52.94 e	24.67 b	1.13 a
	Ińsko Lake District	37.06 c	42.78 d	0.78 a
	mean	45.00 C	33.73 B	0.96 A
Fumaric acid	Uznam–Wolin	4.04 b	21.56 d	0.34 a
	Ińsko Lake District	5.11 bc	5.78 c	0.12 a
	mean	4.58 B	13.67 C	0.23 A
Citric acid	Uznam–Wolin	n.d.	112.26 c	11.51 b
	Ińsko Lake District	2.04 a	128.34 d	10.96 b
	mean	2.04 A	120.30 C	11.24 B
Succinic acid	Uznam–Wolin	3.19 c	n.d.	0.72 a
	Ińsko Lake District	1.17 b	n.d.	0.68 a
	mean	2.18 B	n.d.	0.70 A
Cinnamic acid	Uznam–Wolin	0.23 b	1.10 c	n.d.
	Ińsko Lake District	0.09 a	2.58 d	n.d.
	mean	0.16 A	1.84 B	
Caffeic acid	Uznam–Wolin	0.18 a	n.d.	0.35 a
	Ińsko Lake District	1.33 c	n.d.	0.77 b
	mean	0.76 A	n.d.	0.56 A
Chlorogenic acid	Uznam–Wolin	12.55 f	1.25 d	0.17 a
	Ińsko Lake District	9.34 e	1.06 c	0.78 b
	mean	10.95 C	1.16 B	0.48 A
Ferulic acid	Uznam–Wolin	1.04 c	n.d.	0.21 a
	Ińsko Lake District	0.78 b	n.d.	0.17 a
	mean	0.91 B	n.d.	0.19 A
p-Coumaric acid	Uznam–Wolin	78.36 b	n.d.	N.D.
	Ińsko Lake District	30.11 a	n.d.	N.D.
	mean	54.24	n.d.	
Galic acid	Uznam–Wolin	8.21 c	0.98 b	17.83 e
	Ińsko Lake District	1.09 b	0.20 a	11.28 d
	mean	4.65	0.59	14.56
Salicylic acid	Uznam–Wolin	N.D.	0.12 a	1.79 c
	Ińsko Lake District	N.D.	0.09 a	1.33 b
	mean		0.11 A	1.56 B
Vanillic acid	Uznam–Wolin	18.33 c	n.d.	3.05 b
	Ińsko Lake District	20.58 d	n.d.	1.17 a
	mean	19.46 B	n.d.	2.11 A
Quercetin	Uznam–Wolin	2.38 c	1.45 ab	6.77 d
	Ińsko Lake District	1.85 b	1.11 a	9.12 e
	mean	2.12 B	1.28 A	7.95 C
Catechin	Uznam–Wolin	4.90 a	12.59 b	25.61 d
	Ińsko Lake District	12.31 b	17.33 c	30.54 e
	mean	8.61 A	14.96 B	28.08 C

* Means marked with the same letters do not differ significantly at the significance level of *p* ≤ 0.05. Uppercase letters in the columns (vertical comparison) indicate comparisons between locations (Uznam–Wolin and Ińsko Lake District) for a given mushroom species. Uppercase letters in the last row of the table refer to comparisons of mean values between mushroom species. Lowercase letters indicate statistically significant (or non-significant) differences in the interaction between the examined factors.

**Table 4:** Antioxidant activity of mushroom extracts depending on species and collection site.

		Species of Mushroom		
	Location (Physical-Geographical Region)	*Boletus edulis*	*Imleria badia*	*Leccinum scabrum*
DPPH· (mmol TE/100 g)	Uznam–Wolin	28.9 c *	40.1 d	10.6 a
	Ińsko Lake District	26.9 c	54.5 e	17.8 b
	mean	27.9 B	47.3 C	14.2 A
ABTS·+ (mmol TE/100 g)	Uznam–Wolin	12.4 ab	29.8 c	35.7 e
	Ińsko Lake District	10.9 a	14.6 b	32.1 d
	mean	11.7 A	22.2 B	33.9 C
FRAP (mmol Fe^2+^/100 g)	Uznam–Wolin	22.9 b	43.1 d	58.3 f
	Ińsko Lake District	16.4 a	27.9 c	49.0 e
	mean	19.7 A	35.5 B	53.7 C

* Means marked with the same letters do not differ significantly at the significance level of *p* ≤ 0.05. Uppercase letters in the columns (vertical comparison) indicate comparisons between locations (Uznam–Wolin and Ińsko Lake District) for a given mushroom species. Uppercase letters in the last row of the table refer to comparisons of mean values between mushroom species. Lowercase letters indicate statistically significant (or non-significant) differences in the interaction between the examined factors.

**Table 5:** Total heavy metal content in soils and mushrooms from different physical-geographical regions [mg/kg].

		Species of Mushroom		
	Location (Physical-Geographical Region)	*Boletus edulis*	*Imleria badia*	*Leccinum scabrum*
Cd soil	Uznam–Wolin	1.46 a *	1.62 a	1.71 a
	Ińsko Lake District	1.14 a	1.75 a	1.26 a
	mean	1.30 A	1.68 A	1.49 A
mushrooms	Uznam–Wolin	2.83 d	0.74 b	0.31 ab
	Ińsko Lake District	2.64 d	0.11 a	1.93 c
	mean	2.74 C	0.43 A	1.12 B
Pb soil	Uznam–Wolin	12.59 a	25.65 a	27.58 a
	Ińsko Lake District	30.96 a	19.52 a	17.53 a
	mean	21.77 A	22.58 A	22.56 A
mushrooms	Uznam–Wolin	1.58 b	0.61 a	0.87 a
	Ińsko Lake District	1.64 b	2.67 d	2.22 c
	mean	1.61 A	1.64 A	1.55 A
Ni soil	Uznam–Wolin	4.34 a	7.84 b	6.92 b
	Ińsko Lake District	7.75 b	7.22 b	6.48 ab
	mean	6.04 A	7.53 A	7.70 A
mushrooms	Uznam–Wolin	3.92 b	3.07 a	3.09 a
	Ińsko Lake District	5.89 c	3.34 a	3.26 a
	mean	4.91 B	3.21 A	3.18 A

* Means marked with the same letters do not differ significantly at the significance level of *p* ≤ 0.05. Uppercase letters in the columns (vertical comparison) indicate comparisons between locations (Uznam–Wolin and Ińsko Lake District) for a given mushroom species. Uppercase letters in the last row of the table refer to comparisons of mean values between mushroom species. Lowercase letters indicate statistically significant (or non-significant) differences in the interaction between the examined factors.

**Table 6:** Bioconcentration factors (BCF) of heavy metals in selected fungal species by region.

		Species of Mushroom		
	Location (Physical-Geographical Region)	*Boletus edulis*	*Imleria badia*	*Leccinum scabrum*
Ni	Uznam–Wolin	0.9	0.4	0.4
	Ińsko Lake District	0.8	0.5	0.5
	mean	0.8	0.4	0.4
Cd	Uznam–Wolin	1.9	0.5	0.2
	Ińsko Lake District	2.3	0.1	1.5
	mean	2.1	0.3	0.8
Pb	Uznam–Wolin	0.1	0.02	0.03
	Ińsko Lake District	0.1	0.1	0.1
	mean	0.1	0.1	0.1

## Data Availability

The samples and any additional research data are available from the authors on request.
